# Interaction of the epigenetic integrator UHRF1 with the MYST domain of TIP60 inside the cell

**DOI:** 10.1186/s13046-017-0659-1

**Published:** 2017-12-21

**Authors:** Waseem Ashraf, Christian Bronner, Liliyana Zaayter, Tanveer Ahmad, Ludovic Richert, Mahmoud Alhosin, Abdulkhaleg Ibrahim, Ali Hamiche, Yves Mely, Marc Mousli

**Affiliations:** 1 Laboratoire de Biophotonique et Pharmacologie, UMR 7213 CNRS, Faculté de Pharmacie, Université de Strasbourg, 74, Route du Rhin, 67401 Illkirch Cedex, France; 2 Institut de Génétique et de Biologie Moléculaire et Cellulaire, INSERM U964 CNRS UMR 7104, Université de Strasbourg, Illkirch, France; 3 Department of Biochemistry, Faculty of Science, King Abdulaziz University, Jeddah, Saudi Arabia; 4 Cancer Metabolism and Epigenetic Unit, Faculty of Science, King Abdulaziz University, Jeddah, Saudi Arabia; 5 BioTechnology Research Center (BTRC), Tripoli, Libya

**Keywords:** Cancer, Epigenetics, Fluorescence lifetime imaging microscopy (FLIM), Fluorescence resonance energy transfer (FRET), Protein-protein interaction, TIP60, UHRF1, Cell cycle

## Abstract

**Background:**

The nuclear epigenetic integrator UHRF1 is known to play a key role with DNMT1 in maintaining the DNA methylation patterns during cell division. Among UHRF1 partners, TIP60 takes part in epigenetic regulations through its acetyltransferase activity. Both proteins are involved in multiple cellular functions such as chromatin remodeling, DNA damage repair and regulation of stability and activity of other proteins. The aim of this work was to investigate the interaction between UHRF1 and TIP60 in order to elucidate the dialogue between these two proteins.

**Methods:**

Biochemical (immunoprecipitation and pull-down assays) and microscopic (confocal and fluorescence lifetime imaging microscopy; FLIM) techniques were used to analyze the interaction between TIP60 and UHRF1 in vitro and in vivo. Global methylation levels were assessed by using a specific kit. The results were statistically analyzed using Graphpad prism and Origin.

**Results:**

Our study shows that UHRF1, TIP60 and DNMT1 were found in the same epigenetic macro-molecular complex. In vitro pull-down assay showed that deletion of either the zinc finger in MYST domain or deletion of whole MYST domain from TIP60 significantly reduced its interaction with UHRF1. Confocal and FLIM microscopy showed that UHRF1 co-localized with TIP60 in the nucleus and confirmed that both proteins interacted together through the MYST domain of TIP60. Moreover, overexpression of TIP60 reduced the DNA methylation levels in HeLa cells along with downregulation of UHRF1 and DNMT1.

**Conclusion:**

Our data demonstrate for the first time that TIP60 through its MYST domain directly interacts with UHRF1 which might be of high interest for the development of novel oncogenic inhibitors targeting this interaction.

## Background

Ubiquitin-like containing PHD and RING Finger domains 1 (UHRF1) is a multi-domain nuclear protein that plays an important role in epigenetics through the maintenance of DNA methylation patterns during DNA replication [1, 2]. UHRF1 senses hemi-methylated strand through its SRA domain and then recruits the DNA methyltransferase 1 (DNMT1) to duplicate the methylation patterns on the newly formed daughter strand [3–5]. Besides the readout of DNA methylation marks, UHRF1 also reads histone post-translational modifications (H3K9me2/3) via its tandem tudor and PHD domains and ubiquitinylates histone H3 at lysine 23 by its C-terminal RING domain [6–9]. UHRF1 is highly expressed in proliferating cells as compared with differentiated cells and its level peaks during the G1/S phase transition and G2/M phase of the cell cycle [1, 10]. In cancer cells, UHRF1 is mostly up-regulated and its levels are maintained constant throughout the cell cycle. The high levels of UHRF1 found in variety of cancers are often correlated to the epigenetically silencing of tumor suppressor genes, poor prognosis and aggressiveness of the tumor [11–15]. UHRF1 is stabilized in the cells by its association with the ubiquitin specific protease 7 (USP7 or HAUSP) which prevents the proteasomal degradation of UHRF1 [16]. UHRF1 also plays an important role in regulating the stability and functions of other proteins such as DNMT1, promyelocytic leukemia protein (PML) and p53 through its interaction with other proteins such as the Tat-interacting protein 60 kDa (TIP60), USP7 and histone deacetylase 1 (HDAC1) [17–20]. UHRF1 and TIP60 were shown to be in the same epigenetic complex and to play an important role in regulating the stability and activity of DNMT1 [19, 21]. DNMT1 is acetylated by TIP60 which allows UHRF1 to ubiquitinylate DNMT1 and induce its down-regulation [19].

**Fig. 1:**
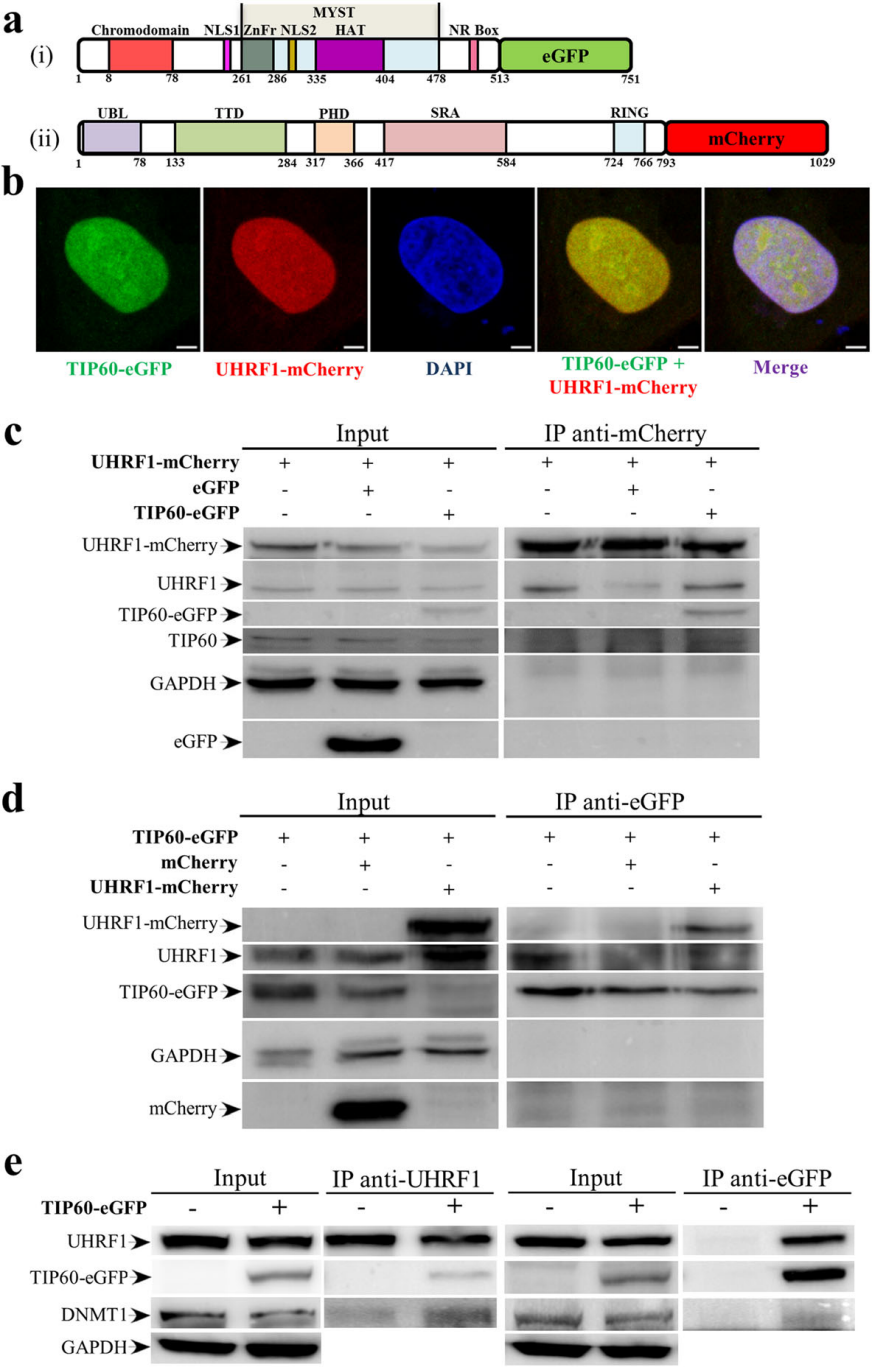
TIP60 interacts with UHRF1 and DNMT1 in HeLa cells. **a** Schematic diagram of TIP60 wild type tagged with eGFP (i) and UHRF1 tagged with mCherry (ii) at their C-terminus. **b** Transfection of TIP60-eGFP and UHRF1-mCherry in the nucleus of HeLa cells. White bar indicates size of 5 μm. **c** Immunoprecipitation of UHRF1-mCherry with anti-mCherry antibody co-immunoprecipitating exogenous TIP60-eGFP and endogenous TIP60. **d** Reciprocal immunoprecipitation of TIP60-eGFP with anti-eGFP antibody co-immunoprecipitating exogenous UHRF1-mCherry and endogenous UHRF1. **e** DNMT1 co-immunoprecipitate with UHRF1 and TIP60-eGFP using anti-UHRF1 and anti-eGFP antibody respectively

TIP60, initially identified as a partner of the HIV-1 Tat protein, is an evolutionary conserved and ubiquitously expressed acetyltransferase of the MYST family [22–25]. The TIP60 protein contains several domains (Fig. 1a, (i)), including a chromodomain and MYST domain endowed with acetyltransferase activity. Through these domains, TIP60 acetylates both histone and non-histone proteins. Tip60 also interacts with androgenic receptors and transcription factors and is involved in a variety of cellular activities including DNA damage response, chromatin remodeling, gene transcription, cell cycle regulation and apoptosis [26–29]. It also mediates the progression of the cell cycle by facilitating the G1/S phase transition, maintaining the genome integrity during the G1 and S phase and ensuring the faithful chromatin segregation during the M phase [30–33]. TIP60 also plays a role in regulating the activities of p53 in an acetylation-dependent and independent manner [18]. TIP60 mediated K120 acetylation in DNA binding region of p53 is necessary for the induction of apoptosis through Bcl 2-associated X protein (BAX) and p53 upregulated modulator of apoptosis (PUMA) pathway. The knockdown of TIP60 has been shown to abrogate the p21-induced cell cycle arrest after the activation of the tumor suppressor gene p53 in response to DNA damage [34–36]. Of note, UHRF1 by its direct interaction with TIP60 through the SRA and RING domains is thought to perturb the association between TIP60 and p53, preventing this latter from an acetylation-dependent activation and antitumor response [18]. Thus, a new anticancer strategy would be to restore p53 function by hindering UHRF1 to interact with TIP60. Although, the literature [18, 21] clearly suggests the occurrence of such an interaction in cells, its final demonstration is still lacking.

In order to further explore this interaction in cells and identify its determinants, we performed Fluoresecence Lifetime Imaging Microscopy (FLIM) experiments to demonstrate that UHRF1 and TIP60 physically interacts inside the cells. Through the use of deletion mutants of TIP60, we identified the key role of the MYST domain in its interaction with the UHRF1. This interaction also occurs in the S phase of the cell cycle during DNA replication.

## Methods

### Cell cultures

HeLa cells (ATCC, CCL-2 Amp, HeLa; Cervical Adenocarcinoma; Human) were cultured in Dulbecco’s modified Eagle’s medium (DMEM + GlutaMAX, Gibco, Lifetech, France) supplemented with 10% of heat inactivated fetal bovine serum and mixture of penicillin (100 U/ml) and streptomycin (100 U/ml) (penicillin/streptomycin: Invitrogen Corporation Pontoise, France) at 37 °C in 5% CO_2_. Transfection of the plasmids in HeLa cells was carried by the jetPEI™ reagent (Life Technologies, Saint Aubin, France) according to the manufacturer’s protocol.

### Plasmid constructs

For HeLa cell transfection, UHRF1 was cloned into pCMV-mCherry vector to express UHRF1-mCherry protein while the TIP60 wild-type and mutants were cloned into a pEGFP-N1 plasmid to express eGFP-labeled TIP60 proteins in cells. For protein purification, UHRF1 was cloned into pGEX-4 T-1 to get the recombinant GST-UHRF1 fusion protein as described in [1]. For in vitro studies, TIP60 wild-type (TIP60-WT) and mutant TIP60 proteins were cloned into pET15b vector with XhoI and BamHI restriction sites to purify His tagged TIP60WT/mutants from bacteria.

### Antibodies

Antibodies used in this study include the mouse monoclonal anti-UHRF1 engineered as described previously [1], mouse monoclonal anti-DNMT1 (Stressgen Canada), rabbit polyclonal anti-TIP60 (Genetex GTX 112197), rabbit polyclonal anti-mCherry (Genetex GTX59788), mouse monoclonal anti-eGFP (Thermo Fisher Scientific A-11120 & Proteintech 66,002–1-Ig), and mouse monoclonal anti-GAPDH (Merck Millipore MAB 374). Mouse monoclonal anti-His and mouse monoclonal anti-GST antibodies were engineered in our core facilities (IGBMC, Illkirch, France).

### Protein purification and pull-down assays

For protein purification, the plasmids were transfected in BL21 cells and cells were allowed to grow at 37 °C until the absorbance of the culture reached 0.5–0.6. Expression of the proteins was induced by the addition of 1 mM isopropyl-1-thio-β-D-galactopyranoside (IPTG) and the cells were further incubated at 25 °C for 4 h before collecting the proteins. GST-tagged UHRF1 protein was purified from the cell lysate using Glutathione Sepharose 4B beads (GE Healthcare Life Sciences 17–0756-05) while the His-tagged wild-type and mutant TIP60 proteins were purified using Ni-NTA agarose beads (Qiagen 30,230) in appropriate buffers. Wild-type and mutant TIP60 proteins were immobilized on the Ni-NTA agarose beads and equal quantity of GST-UHRF1 was added in PBS containing 30 mM imidazole and 0.1% triton to study protein-protein interaction. The immobilized beads were washed five times before being analyzed by SDS-PAGE.

### Fluorescence lifetime imaging microscopy (FLIM)

For FLIM measurements, 10^5^ cells were seeded in a μ-dish 35 mm, glass bottom grid-50 (Ibidi 81,148) wells and were co-transfected with 0.75 μg TIP60-eGFP and 0.75 μg UHRF1-mCherry plasmids by using jetPEI™ reagent as described in manufacturer’s protocol. After 24 h of transfection, cells were incubated for 20 min with 10 μM 5-ethynyl-2′-deoxyuridine (EdU) containing media before fixation with 3.7% paraformaldehyde. After fixation, cells were analyzed with a homemade two-photon excitation scanning microscope based on an Olympus IX70 inverted microscope with an Olympus 60X 1.2 NA water immersion objective operating in the descanned fluorescence collection mode as described [37]. Two-photon excitation at 930 nm was provided by an Insight DeepSee laser (Spectra Physics). Photons were collected using a short pass filter with a cut-off wavelength of 680 nm (F75–680, AHF, Germany) and a band-pass filter of 520 ± 17 nm (F37–520, AHF, Germany). The fluorescence was directed to a fiber coupled APD (SPCM-AQR-14-FC, Perkin Elmer), which was connected to a time-correlated single photon counting module (SPC830, Becker & Hickl, Germany). FLIM data were analyzed using the SPCImage v 4.0.6 (Becker & Hickel) software. The Förster resonance energy transfer (FRET) efficiency was calculated according to E = 1- (τ_DA_/τ_D_), where τ_DA_ is lifetime of donor (eGFP) in the presence of acceptor (mCherry) and τ_D_ is the lifetime of donor in the absence of acceptor.

### Confocal microscopy

The cells imaged by FLIM were also imaged by confocal microscopy. The same cells could be imaged by both techniques, by locating the cells with the help of coordinates on the ibidi well. Prior to confocal microscopy the cells in S phase were labeled with the Click-iT® EdU Alexa Fluor® 647 Imaging Kit (Thermo Fisher Scientific USA C10340) according to the manufacturer’s protocol. For transfection and localization analysis, cells were co-transfected with TIP60-eGFP WT/mutants and UHRF1-mCherry and were labeled with DAPI after fixation to stain the nucleus. All samples were imaged with a Leica SPE equipped with a 63× 1.4NA oil immersion objective (HXC PL APO 63×/1.40 OIL CS). The images were further processed with Image J software.

### Immunoprecipitation and western blotting

For Western blot, cells were harvested 24 h post-transfection by mild trypsinization. After washing with PBS, cells were lysed by ice cold lysis buffer 10 mM Tris-HCl pH 7.5, 150 mM NaCl, 1 mM EDTA and 1% NP40 supplemented with protease inhibitors (complete mini EDTA free protease inhibitor cocktail tablets, Roche Germany 11,836,170,001). Cell lysates (40 μg of the protein) were loaded onto 10% SDS-PAGE gels after denaturation for 5 min in Laemmli sample buffer (Bio-Rad Laboratories USA 1610747). The proteins were identified by anti-UHRF1, anti-eGFP, anti-DNMT1 and anti-GAPDH antibodies with overnight incubation at 4 °C. Primary antibodies were labeled with secondary anti-mouse (Promega, W402B) or anti-rabbit antibodies (Promega, W401B) conjugated with horseradish peroxidase and were visualized with the chemiluminescent ECL system (Clarity™ ECL western blotting substrate, Biorad, 170–5060) on an Image Quant LAS 4000 apparatus. Images were analyzed using the Image Studio Lite (Li-Core Biosciences, USA). For co-immunoprecipitation, the cells were collected and lysed by freeze shock and sonication in PBS supplemented with protease inhibitor cocktail tablet. A fraction of 40 μg of protein from each lysate was saved to serve as input control while 800 μg to 1 mg of protein lysate was incubated with appropriate antibodies for 4 h at 4 °C for subsequent immunoprecipitation. After washing and equilibration, 50 μL of Dynabeads® Protein A (Thermo Fisher Scientific Norway 10002D) were added to the lysate-antibody mixture and incubated for 1 h at 4 °C. Beads were collected later and washed five times in lysis buffer. They were then resuspended in Laemmli sample buffer (Bio-Rad Laboratories, USA). Proteins denatured by heating at 95 °C for 5 min were analyzed through Western blotting.

### Global DNA Methylation analysis

HeLa cells were transiently transfected with TIP60-eGFP and mutants and were analyzed for global methylation levels by using Sigma’s Imprint® Methylated DNA Quantification Kit Sigma-Aldrich). Briefly DNA was extracted from the cells using QIAamp® DNA Kit (Qiagen) and 200 ng of purified DNA were used for global DNA methylation level analysis according to the manufacturer’s protocol.

### Statistical analysis

The results were statistically analyzed using GraphPadPrism (version 5.04) and Origin (version 8.6).

## Results

### UHRF1 and TIP60 interaction inside the cells

In order to study the interaction between TIP60 and UHRF1, we expressed eGFP-tagged TIP60 (Fig. 1a *,* (i)) and mCherry-tagged UHRF1 (Fig. 1a *,* (ii)) in HeLa cells. The two proteins were expressed and co-localized with DAPI inside the nucleus of HeLa cells as seen by the merge (Fig. 1b). The interaction between UHRF1 and TIP60 proteins was assessed in vitro by co-immunoprecipitation experiments. Immunoprecipitating UHRF1-mCherry by using anti-mCherry antibody led to the co-immunoprecipitation of both endogenous TIP60 and exogenous TIP60-eGFP while free eGFP which was co-transfected with UHRF1-mCherry did not co-immunoprecipitate with it (Fig. 1c). This shows specific interaction of UHRF1-mCherry with endogenous TIP60 and exogenous TIP60-eGFP. Similarly, reciprocal co-immunoprecipitation experiments were performed by immunoprecipitating TIP60-eGFP with anti-eGFP antibody in cells (Fig. 1d). Immunoprecipitation of TIP60-eGFP led to co-immunoprecipitation of UHRF1-mCherry and endogenous UHRF1 while it did not immunoprecipitate free mCherry suggesting specific interaction between UHRF1 and TIP60 in the cells. Therefore, we can assume that tagged proteins correctly localize in the nucleus of HeLa cells and can mimic the interaction pattern of endogenous proteins. It is interesting to note that UHRF1-mCherry co-expression resulted in lower levels of TIP60-eGFP recombinant protein (Fig. 1d) as compared with cells transfected with TIP60-eGFP or co-transfected with mCherry alone.

LikeTIP60, DNMT1 has also been reported to be associated with UHRF1 in the same protein complex [21]. So, in order to check the presence of DNMT1 in UHRF1/TIP60 complex, we also performed co-immunoprecipitation experiments. DNMT1 co-immunoprecipitated with the UHRF1 in normal HeLa cells or cells with overexpressed TIP60-eGFP (Fig. 1e). Overexpressed TIP60-eGFP also interacted with endogenous DNMT1 as DNMT1 co-immunoprecipitated with TIP60-eGFP along with UHRF1 showing the presence of the three proteins together in the same complex (Fig. 1e). This supports that the tag of TIP60-eGFP does not hinder it to adequately interact with its partners like DNMT1.

However, the results obtained with immunoprecipitation cannot confirm the interaction of proteins in vivo and do not explain the presence or absence of a close dialogue between the two proteins inside the cell.

**Fig. 2:**
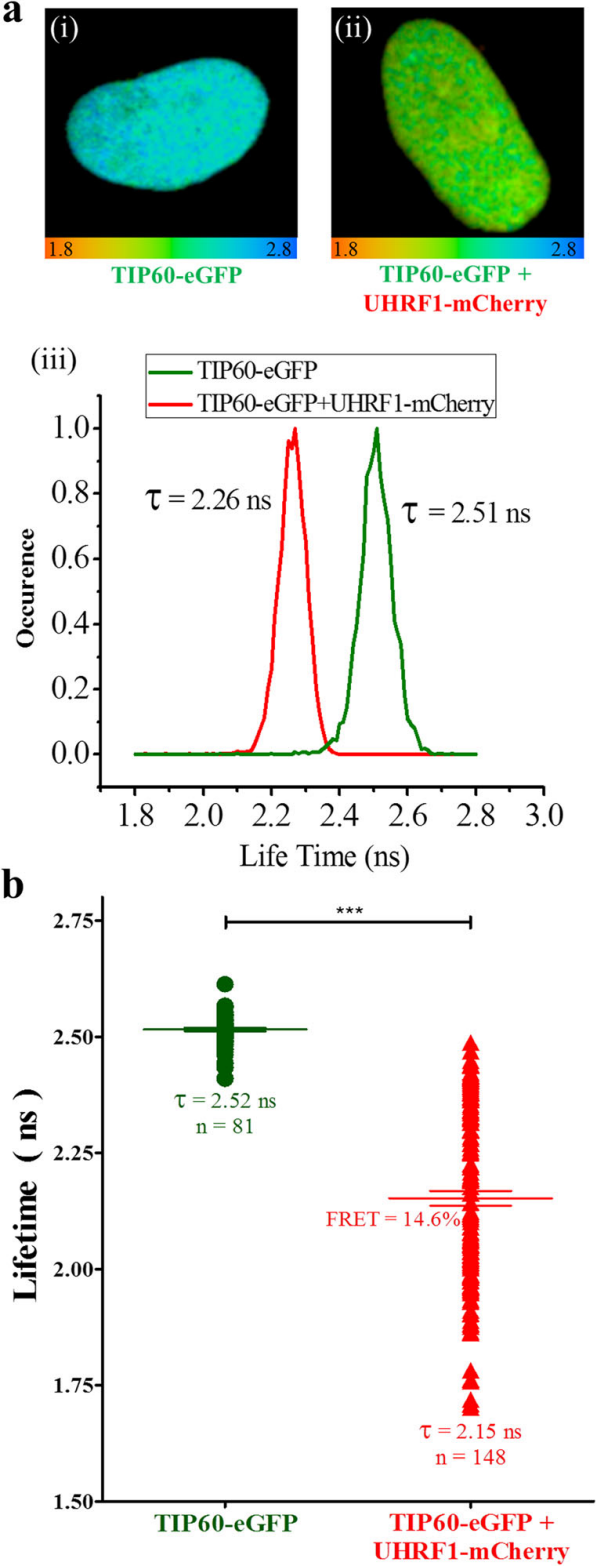
Interaction of TIP60-eGFP with UHRF1-mCherry evidenced by FRET-FLIM. **a** 25 μm × 25 μm FLIM images of HeLa cells transfected with TIP60eGFP (i) or co-transfected with TIP60-eGFP and UHRF1-mCherry (ii) and lifetime distribution curve (iii). Color coded images indicate the fluorescence lifetime of TIP60-eGFP at each pixel. Color scale codes for lifetimes ranging from 1.8 ns (red) to 2.8 ns (blue). **b** Fluorescence lifetimes in TIP60-eGFP () and TIP60-eGFP + UHRF1-mCherry co-transfected cells (). Values are means ± SEM from five independent experiments. For statistical analysis, a Student’s *t*-test was performed (*** *P* < 0.001)

Therefore, we studied the interaction between UHRF1 and TIP60 in cells using the FLIM-FRET technique which allows monitoring of very close contact (< 10 nm) between two proteins inside a cell. TIP60-eGFP served as the FRET pair donor because of the mono-exponential decay and high quantum yield of eGFP while the UHRF1-mCherry served as the FRET pair acceptor in these experiments as the absorption spectrum of mCherry falls in the emission spectrum of the eGFP. FRET occurs only when the two fluorophores are in close proximity to each other and can be unambiguously evidenced by a decrease of lifetime of the donor. By using FLIM microscopy, the lifetime of eGFP is calculated and color coded in each pixel of the image. The red to blue color covers lifetime ranging from 1.8 ns to 2.8 ns. FLIM images were recorded for TIP60-eGFP transfected cells (Fig. 2a, (i)) and cells co-transfected with TIP60-eGFP and UHRF1-mCherry (Fig. 2a, (ii)). The resulting distributions of fluorescent lifetimes are given in (Fig. 2a, (iii)). The average lifetime of TIP60-eGFP was 2.52 ± 0.01 ns in the cells transfected with TIP60-eGFP alone (Fig. 2b) or co-transfected with free mCherry (data not shown). However, the lifetime of eGFP was significantly reduced when TIP60-eGFP was co-transfected with UHRF1-mCherry in 1:1 ratio (Fig. 2b). The average lifetime of eGFP in co-transfected cells was 2.15 ± 0.02 ns, which corresponds to a mean FRET efficiency of 14.3 ± 0.6% (Fig. 2b). Altogether, these findings demonstrate that TIP60-eGFP interacts with UHRF1-mCherry in HeLa cells.

### UHRF1 and TIP60 interaction occurs during S phase of the cell cycle

**Fig. 3:**
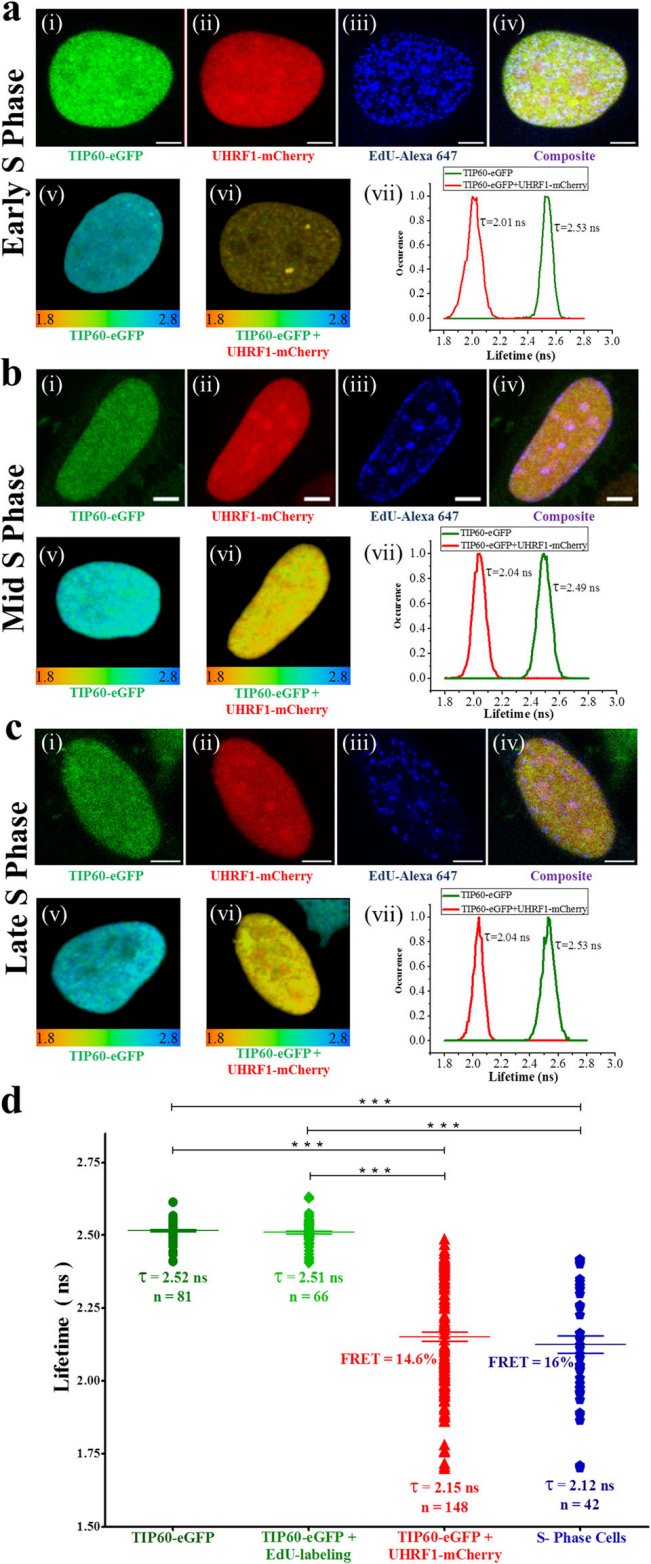
Interaction between TIP60-eGFP and UHRF1-mCherry in S phase of cell cycle. **a-c** TIP60-eGFP interaction with UHRF1-mCherry in early, mid and late S phases of cell cycle, respectively. Confocal images of cells labeled with TIP60-eGFP, UHRF1-mCherry, EdU-Alexa 647 and merge, respectively (i - iv). The white bar indicates size of 5 μm. 25 μm × 25 μm FLIM images of HeLa cells transfected with TIP60-eGFP (v) or co-transfected with TIP60-eGFP and UHRF1-mCherry (vi) and lifetime distribution curves of the respected cells (vii). Color scale codes for lifetimes ranging from 1.8 ns (red) to 2.8 ns (blue). **d** Fluorescence lifetime distributions of TIP60-eGFP (), TIP60-eGFP EdU labeled cells (), total TIP60-eGFP + UHRF1-mCherry co-transfected cells () and co-transfected cells in S-phase of cell cycle (). Values are means ± SEM from five independent experiments. For statistical analysis, a Student’s *t*-test was performed (*** *P* < 0.001)

UHRF1 localization and its association with other proteins dynamically changes during the cell cycle. NP95, the murine homologue of UHRF1 associates with PCNA and chromatin in early and mid S phase of cell cycle. Moreover UHRF1 interaction with DNMT1 for maintenance of DNA methylation pattern is also dependent on the S phase of cell cycle and is more pronounced in mid and late S phase of cell cycle [38–40]. Since both UHRF1 and TIP60 are also regulating the DNMT1 levels [19] and TIP60 is also playing important roles during the G1/S phase transition and S phase of the cell cycle [30, 33], we focused on S phase to decipher the interaction between UHRF1 and TIP60. Therefore, we labeled S phase cells undergoing DNA replication with EdU (thymidine analogue) for 15 min before fixation and then, we performed FLIM analysis (Fig. 3). After this, S phase cells were identified using alexa 647 labeling for confocal microscopy study. Different sub-phases of S phase were identified by the characteristic staining of EdU which gets incorporated into the genome at the sites of active replication [41]. Early S phase cells have numerous replication foci in the nucleus as evident by bright and abundant EdU labeling in nucleus of HeLa cells (Fig. 3a). In mid S phase the replication foci are more localized to periphery of nucleus and surrounding the nucleolus (Fig. 3b) while in late S phase, very few irregular replication foci are found in nucleus at heterochromatin regions of genome (Fig. 3c). The lifetime of the TIP60-eGFP was found to be decreased in the different sub-phases of the S phase (Fig. 3a-c). When the average lifetime of TIP60-eGFP in S phase cells was compared to the total cells, it was decreased to 2.12 ± 0.03 ns and the overall FRET efficiency increased to 16.0 ± 1.2% in the S phase positive cells (Fig. 3d). These results confirm UHRF1/ TIP60 interaction during the S phase of cell cycle.

### TIP60 interacts with UHRF1 through its MYST domain

**Fig. 4:**
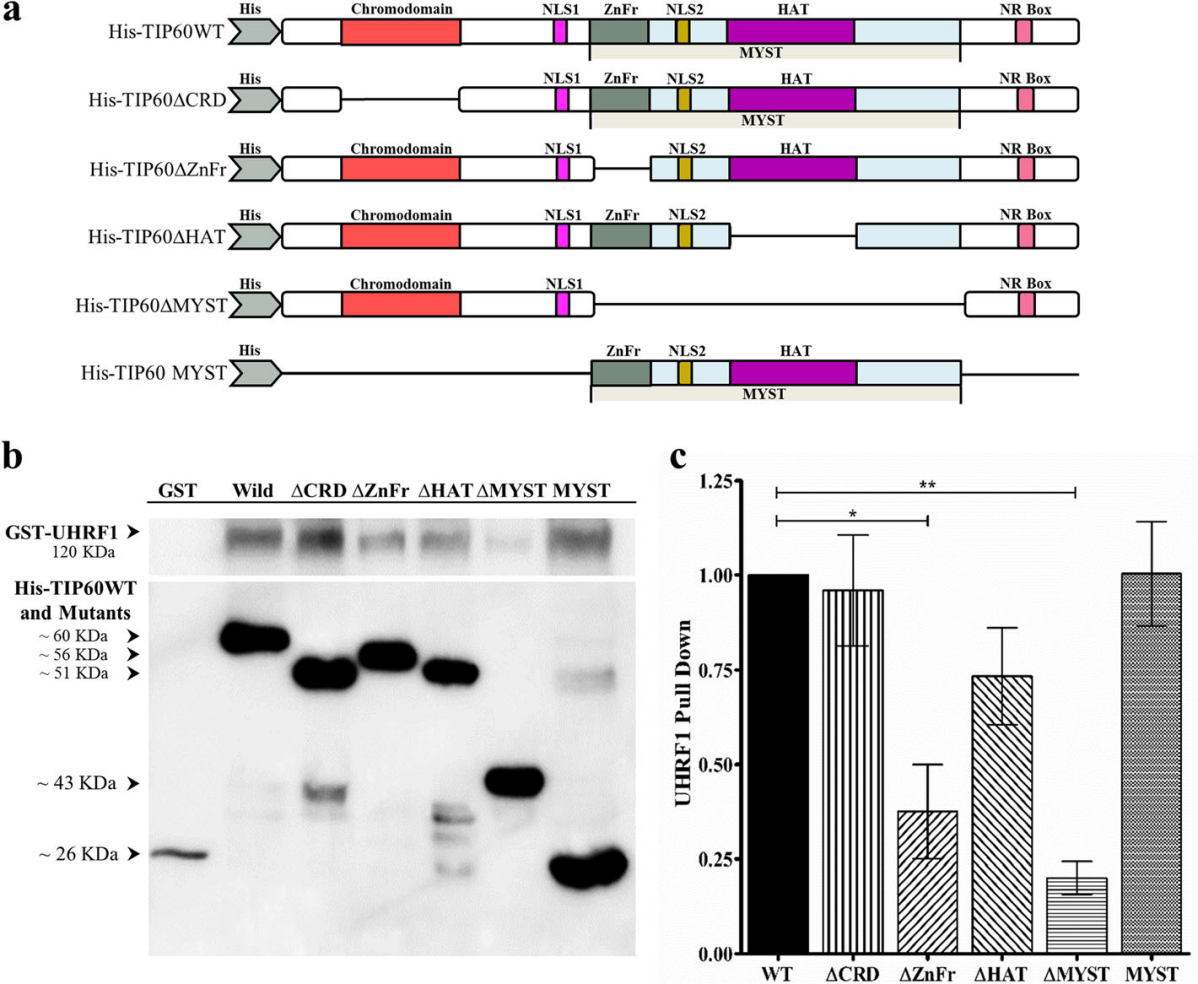
In vitro pull-down analysis between His-TIP60WT/mutants and GST-UHRF1. **a**, Diagram showing His tag TIP60 wild type and mutants. **b** Western blot of in vitro pull-down assay. His tagged TIP60-WT or mutants were immobilized on Ni-NTA beads and incubated with UHRF1-GST. The complex recovered after washing were subjected to SDS PAGE and analyzed by Western blot. **c**. Western blot images were quantified by Image Studio Lite (Li-Core Biosciences USA) and statistically analyzed by using Student’s *t*-test. Values are means ± SEM from three independent experiments (* *P* < 0.05, **P < 0.01)

It is known that UHRF1 interacts with TIP60 through its SRA and RING domains and hinders the association of TIP60 with p53 and K120 acetylation of p53 [18]. However, the TIP60 domain responsible for its interaction with UHRF1 remains to be determined. Therefore, in this study we performed in vitro pull-down assay to identify the domain of TIP60 that is responsible for interaction with UHRF1. For this, we used His-tagged mutants of the TIP60 (Fig. 4a) immobilized on Nickel NTA agarose beads and the GST-UHRF1. We observed that full length UHRF1 interacted with TIP60WT in the presence of 150 mM NaCl (Fig. 4b-c) until 500 mM NaCl (data not shown) supporting a strong interaction between both proteins. Deletion of the TIP60 zinc finger domain or the whole MYST domain significantly reduced its association with GST-UHRF1 in the pull-down assay (Fig. 4b-c). In contrast, deletion of the chromodomain and HAT domains did not significantly affect their interaction with UHRF1. Recombinant TIP60 MYST domain also had a strong association with UHRF1 like the wild type TIP60 protein (Fig. 4b-c) and this interaction was stable up to 1 M NaCl salt concentration (data not shown) predicting the TIP60 MYST domain is playing a key role in this interaction.

**Fig. 5:**
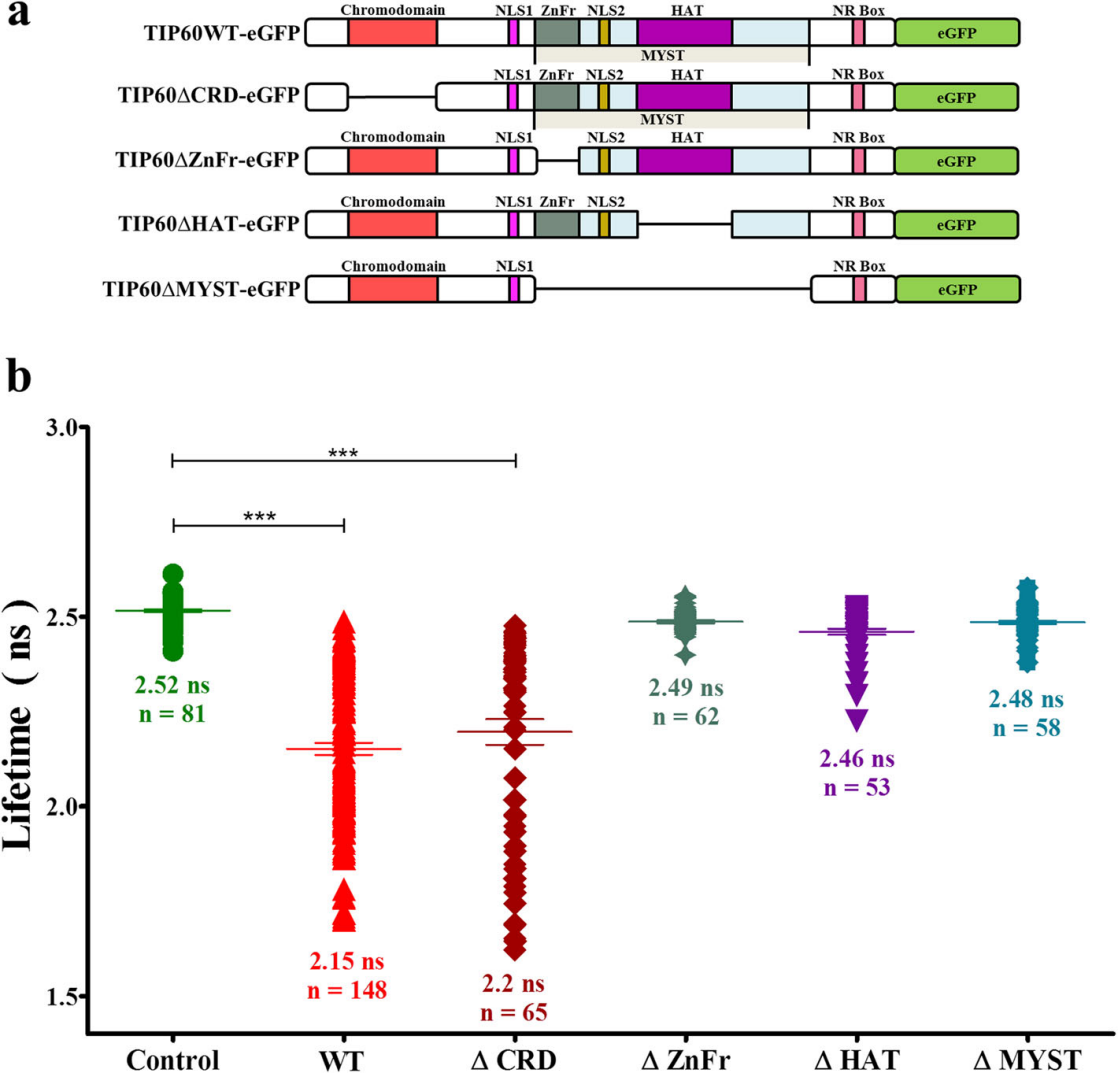
Interaction between TIP60-eGFP WT/mutants and UHRF1 evidenced by FRET-FLIM. **a** Schematic diagram of TIP60WT/mutants tagged with eGFP at the C-terminus. **b** Lifetime distribution of TIP60-eGFP (), TIP60 WT-eGFP + UHRF1-mCherry co-transfected cells (), TIP60 ∆CRD-eGFP + UHRF1-mCherry co-transfected cells (), TIP60 ∆ZnFr-eGFP + UHRF1-mCherry co-transfected cells (), TIP60 ∆HAT-eGFP + UHRF1-mCherry co-transfected cells (), TIP60 ∆MYST-eGFP + UHRF1-mCherry co-transfected cells (). Values are means ± SEM from three to five independent experiments. For statistical analysis, a Student’s *t*-test was performed (*** *P* < 0.001)

**Fig. 6:**
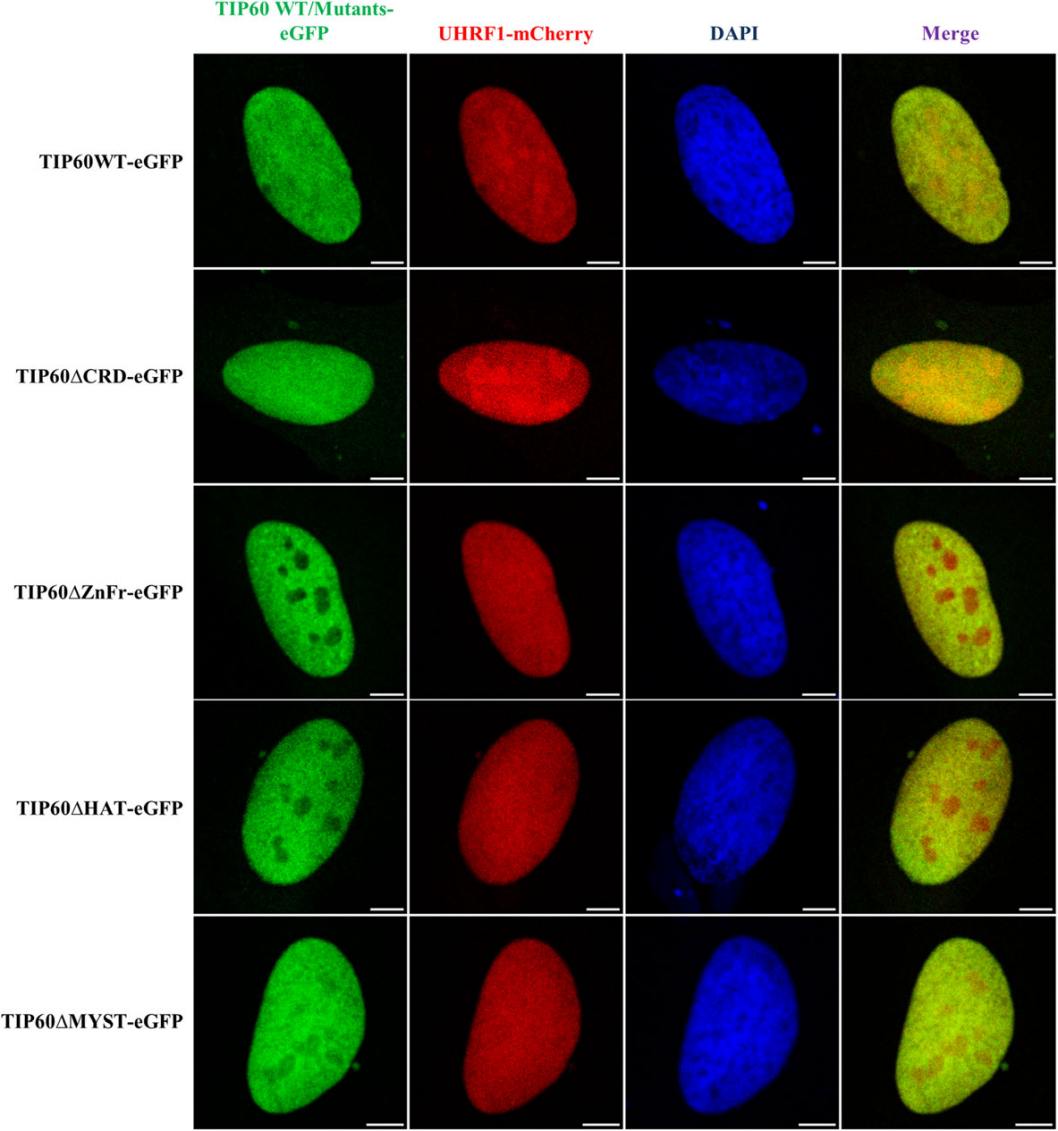
Expression and localization of TIP60 mutants in HeLa cells. Confocal images show the expression and co-localization of TIP60WT-eGFP and mutants with UHRF1-mCherry in the HeLa cells with DAPI labeling. Green panel indicates TIP60 wild type or mutants tagged with eGFP, red panel shows UHRF1-mCherry, blue panel indicates DAPI and merge panel shows the composite of the TIP60-eGFP and UHRF1-mCherry panels. White bar indicates size of 5 μm

The FLIM-FRET technique employing different mutants of TIP60 tagged with eGFP (Fig. 5a) was further used to identify the interacting domain of TIP60 with UHRF1-mCherry inside the nucleus of HeLa cells. TIP60-eGFP wild type and mutants were co-transfected with UHRF1-mCherry and the lifetime of eGFP was measured to assess the interaction. We found that the interaction of TIP60 and UHRF1 was marginally affected by removal of TIP60 chromodomain as the average FRET of TIP60∆CRD-eGFP co-transfected with UHRF1-mCherry was of 12.2 ± 1.3% as compared to 14.3 ± 0.6% for TIP60WT-eGFP (Fig. 5b). All other mutations affecting the MYST domain of TIP60 strongly perturbed the interaction of these mutants with UHRF1. Indeed, the lifetime of TIP60∆ZnFr-eGFP, TIP60∆HAT-eGFP and TIP60∆MYST-eGFP co-transfected with UHRF1-mCherry was 2.49 ± 0.01 ns, 2.46 ± 0.01 ns and 2.49 ± 0.01 ns, respectively which is quite similar to that in control sample with 2.52 ± 0.01 ns (Fig. 5b). To check whether this loss of interaction is not a result of an alteration of subcellular localization, we performed a confocal microscopy analysis of co-transfected HeLa cells. We observed that TIP60WT-eGFP and its mutants including TIP60∆CRD-eGFP, TIP60∆ZnFr-eGFP, TIP60∆HAT-eGFP and TIP60∆MYST-eGFP are localized in the nucleus of HeLa cells (Fig. 6). It is also important to note that TIP60WT and mutants co-localized with UHRF1-mCherry as shown in merge panels and were closely associated to DNA labeled by DAPI. This indicates that the loss of interaction between TIP60∆ZnFr, TIP60∆HAT and TIP60∆MYST with UHRF1 is not due to protein delocalization.

**Fig. 7:**
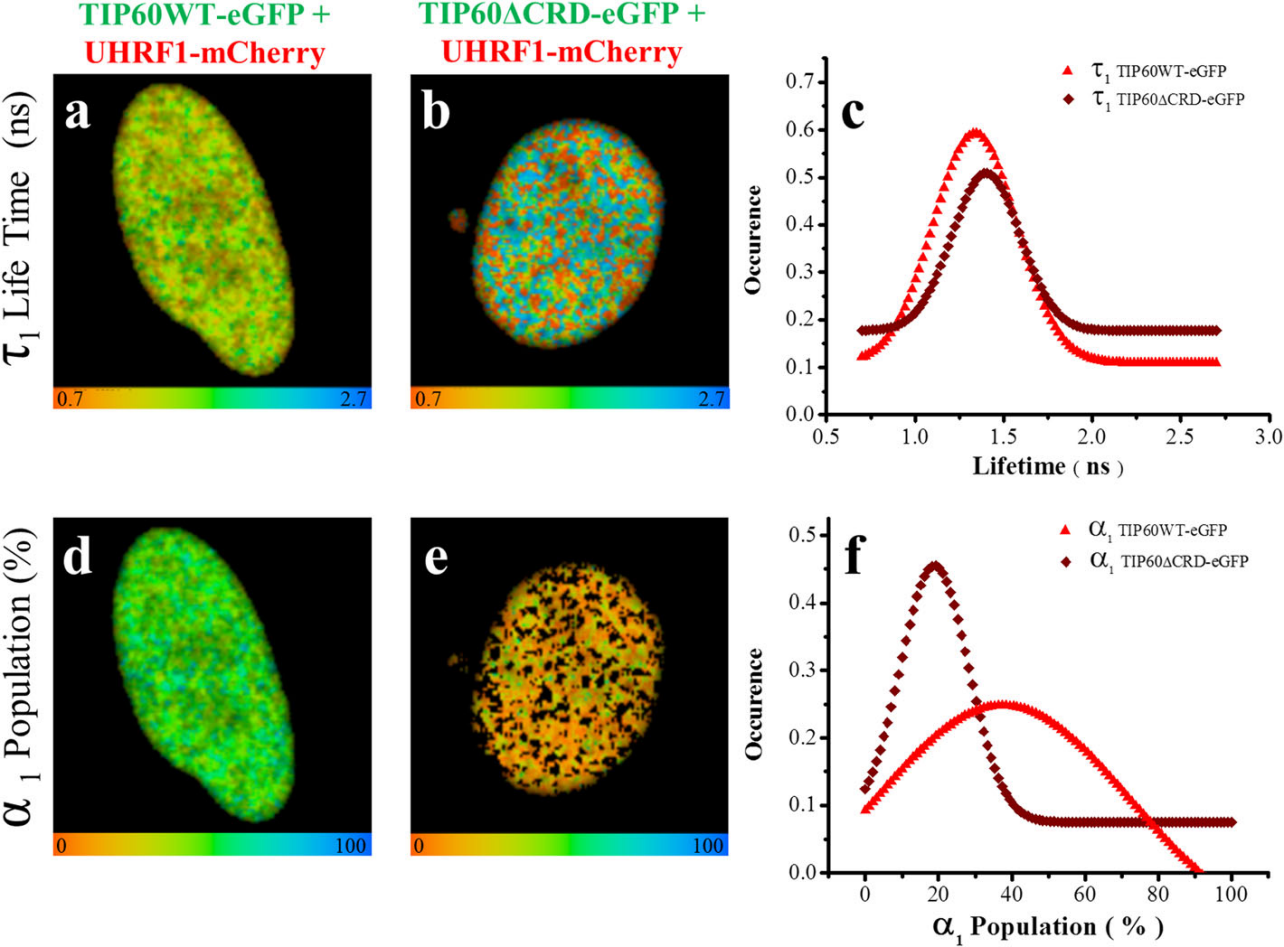
Two component analyses of the fluorescence decays of TIP60WT-eGFP and TIP60∆CRD-eGFP lifetime in presence of UHRF1-mCherry. Fluorescence decays were measured at each pixel for the respective cells by using bi-exponential model. In this model, the long-lived lifetime component (τ_2_) was fixed to the lifetime of Tip6WT-eGFP when it is transfected alone in HeLa cells (2.52 ns). **a** 25 μm × 25 μm FLIM image of the distribution of τ_1_ lifetimes of TIP60WT-eGFP in the presence of UHRF1-mCherry (corresponding to the component undergoing FRET). **b** 25 μm × 25 μm FLIM image of the distribution of τ_1_ lifetimes of TIP60∆CRD-eGFP in the presence of UHRF1-mCherry (corresponding to the component undergoing FRET). Color scale codes for lifetimes ranging from 0.7 ns (red) to 2.7 ns (blue). **c** Distribution of τ_1_ lifetimes of TIP60WT-eGFP and TIP60∆CRD-eGFP transfected cells in presence of UHRF1-mCherry. **d** 25 μm × 25 μm FLIM image of the population α_1_ of TIP60WT-eGFP undergoing FRET in the presence of UHRF1-mCherry. **e** 25 μm × 25 μm FLIM image of the population α_1_ of TIP60∆CRD-eGFP undergoing FRET in the presence of UHRF1-mCherry. Color scale codes for population ranging from 0% (red) to 100% (blue). **f** Distribution of population α_1_ for TIP60WT-eGFP and TIP60∆CRD-eGFP transfected cells in presence of UHRF1-mCherry. Values indicated are from 148 TIP60WT-eGFP and UHRF1-mCherry co-transfected cells from five independent experiments and 65 TIP60∆CRD-eGFP and UHRF1-mCherry co-transfected cells from three independent experiments

In order to check the heterogeneity of lifetime populations in TIP60-eGFP wild type or TIP60∆CRD-eGFP co-transfected cells showing FRET, the FLIM images were also analyzed by a two-component model: F (t) = α_1_e^-t/τ^_1_ + α_2_e^-t/τ^_2_ [37]. This analysis provides the distribution and population of TIP60-eGFP molecules interacting with UHRF1-mCherry (having FRET) and the TIP60-eGFP molecules which are free in nucleus without having interaction with UHRF1-mCherry (having no FRET). The lifetime for the long lifetime component (τ_2_) (having no FRET) was fixed according to the lifetime of eGFP in only TIP60-eGFP transfected samples, while the lifetime (τ_1_) of the short component (having FRET) and the populations of both component (α_1_ and α_2_) were obtained from the fits. The short lifetime component (τ_1_) in TIP60WT-eGFP and TIP60∆CRD-eGFP samples having FRET because of interaction with UHRF1-mCherry are shown in green or warmer color in FLIM images (Fig. 7a-b). The lifetime distribution curves of these FRET components for TIP60WT-eGFP and TIP60∆CRD-eGFP are depicted in Fig. 7c. The mean value of the short lifetime component in TIP60WT-eGFP samples was 1.33 ± 0.01 ns and the average FRET calculated for this component was 45 ± 0.6% indicating close association of TIP60-eGFP with UHRF1-mCherry in HeLa cells. The mean value of the short component in TIP60∆CRD-eGFP was 1.4 ± 0.03 ns and the average FRET calculated for this component was 43 ± 1.1% (Fig. 7c). Though the short lifetime component had almost similar values in TIP60WT-eGFP and TIP60∆CRD-eGFP samples, the values of its corresponding population were different in the two samples as shown in Fig. 7d-e. TIP60WT-eGFP had higher population (α_1_) of interacting short lifetime component as compared to TIP60∆CRD-eGFP as its mean value in TIP60WT-eGFP was 37.5 ± 1.2% while it was 19 ± 0.3% in TIP60∆CRD-eGFP as indicated from their respective distribution curves (Fig. 7f). This shows that TIP60∆CRD-eGFP can interact with UHRF1-mCherry inside the nucleus but with less efficiency than TIP60WT-eGFP.

### TIP60 overexpression down-regulates UHRF1 and DNMT1

**Fig. 8:**
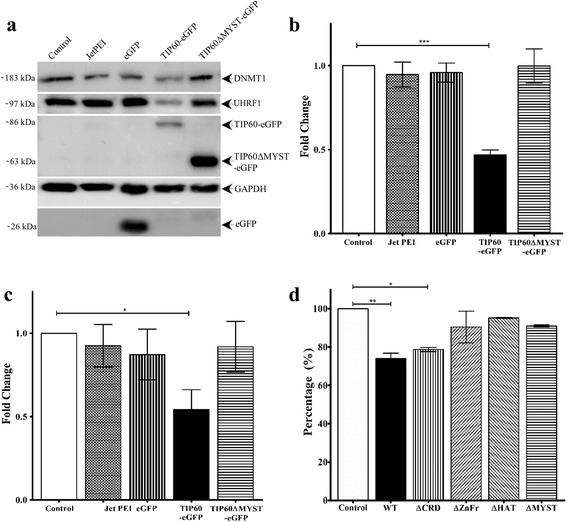
TIP60 overexpression down-regulates its epigenetic partners UHRF1 and DNMT1. TIP60-eGFP was overexpressed in HeLa cells and the effect of this transient overexpression was compared to that of the control HeLa cells, HeLa cells with transfecting agent (JetPEI), HeLa cells with transfection of eGFP alone or TIP60ΔMYST-eGFP. **a** Western blot results showing down-regulation of UHRF1 and DNMT1 in TIP60-eGFP transfected cells. **b** Analysis of effect of TIP60-eGFP overexpression on UHRF1. **c**, Analysis of effect of TIP60-eGFP overexpression on DNMT1. Results indicated are from five independent experiments which are analyzed statistically by Student’s *t*-test (* *P* < 0.05, ** *P* < 0.01, *** *P* < 0.001). **d** Effect of TIP60-eGFP overexpression on global DNA methylation. DNA was extracted from HeLa cells transfected with TIP60WT-eGFP and mutants and the methylation levels were compared to control HeLa cells. Results are indicated from three independent experiments and were analyzed by one-way ANOVA with post-hoc Tukey test. (* *P* < 0.05, ** *P* < 0.01, *** *P* < 0.001)

Down-regulation of TIP60 has been reported in many cancers [42–45] and TIP60 has a well-established role in regulation of DNMT1. So, we investigated the consequences of TIP60-eGFP overexpression on UHRF1 and DNMT1 in HeLa cells in order to decipher the relationship between these epigenetic partners in the tumorigenesis process. Overexpression of TIP60 led to down-regulation of UHRF1 and DNMT1 in HeLa cells (Fig. 8a). UHRF1 levels were significantly reduced in TIP60-eGFP transfected cells as compared to that in untreated control cells, i.e., without any treatment or cells treated with jetPEI or transfected with eGFP alone (Fig. 8b). Similarly, DNMT1 levels were also significantly reduced in cells overexpressing TIP60-eGFP (Fig. 8c). It is interesting to observe that DNMT1 and UHRF1 levels were not affected by the overexpression of TIP60ΔMYST-eGFP in the nucleus which lacks the acetyltransferase domain of TIP60. Further, we also analyzed the effect of TIP60-eGFP overexpression on global DNA methylation levels. In accordance with the decrease in UHRF1 and DNMT1 levels, global DNA methylation also decreased by 26% after overexpression of TIP60WT-eGFP in 24 h of transfection (Fig. 8d). Overexpression of TIP60∆CRD-eGFP also decreased the global DNA methylation by 21% (Fig. 8d), however, over expressing TIP60∆ZnFr-eGFP and TIP60∆MYST-eGFP only lowered the DNA methylation by 9%. Overexpression of TIP60∆HAT-eGFP had minimal effect on global DNA methylation which decreased only by 5% (Fig. 8d). Altogether these results suggest TIP60 as a regulator of DNMT1, UHRF1 and DNA methylation levels through its enzymatic activity.

## Discussion

UHRF1 and TIP60 are part of large protein complexes and their conformation and association with other partners vary with the genomic activity and are regulated during cell cycle [46, 47]. Our results provided evidence for in vivo and in vitro interaction between UHRF1 and TIP60 protein by using the FLIM-FRET technique and pull-down assay. Furthermore, we could also show that MYST domain of TIP60 is playing a major role in its interaction with UHRF1. MYST domain is the conserved part of TIP60 containing a zinc finger involved in protein-protein interaction and a catalytic domain harboring its acetyltransferase activity [47]. In fact, through its MYST domain, TIP60 is able to acetylate both histones and non-histones proteins and regulates the activity of many proteins such as ATM and p53 [25, 36, 48]. Since p53-mediated apoptosis is dependent on its acetylation by TIP60 [35] therefore, interaction of TIP60 through its MYST domain with UHRF1 might impair many cellular functions. This may also explain how overexpressed UHRF1 in cancer negatively regulates the TIP60-p53 interplay in cells by preventing induction of cell cycle arrest and apoptosis. It is interesting to note that although chromodomain is not playing a direct role in its association with UHRF1 as indicated by FLIM and pull-down experiments, its removal can adversely also affect this interaction in vivo. According to two-component model, removal of chromodomain did not have a big impact on the mean lifetime of short component and FRET efficiencies as compared with wild type. However, the population interacting with UHRF1 was drastically reduced when chromodomain was removed from the structure of TIP60. Chromodomain helps TIP60 in reading out the histone marks and its loading to chromatin which may increase the possibility of TIP60 to interact with UHRF1 present in the same complex [49–51].

UHRF1 is a multi-domain protein which is essential for maintaining the DNA methylation during S phase of cell cycle by recruiting DNMT1 to the replication foci where it forms a multi protein complex with PCNA, DNMT1, TIP60, HDAC1, USP7 and other epigenetic partners [38, 52]. TIP60 is also well known for its role in DNA damage response to interstrand cross linkages or double strand breaks as TIP60-mediated H4K16 acetylation promotes DNA damage repair by homologous recombination (HR) pathway which dominates during the S phase of cell cycle [53, 54]. Recently the role of UHRF1 in DNA damage response has also been reported as it identifies interstrand cross linkages and double strand breaks and facilitates DNA damage repair by the same homologous recombination (HR) pathway through interaction with common partners such as FANCD2 and BRCA1 [55–57]. This predicts that UHRF1 and TIP60 may also work together in coherence to facilitate the DNA damage repair during S phase of cell cycle.

TIP60 along with UHRF1 is known to regulate levels of DNMT1 during cell cycle by inducing proteasomal degradation of DNMT1 through TIP60-mediated acetylation and subsequent ubiquitination by UHRF1 [19, 58, 59]. Accordingly, we have observed increased association of DNMT1 with UHRF1 in TIP60-eGFP transfected samples through co-immunoprecipitation experiments confirming the previous findings. DNMT1 is stabilized in cells by its direct association with USP7, a deubiquitinating enzyme which is present in the same complex. It has been recently reported that TIP60 impairs this protective association of USP7 with DNMT1 by acetylation [60]. Besides DNMT1, UHRF1 is also prevented from proteasomal degradation through its association with USP7 [16, 61, 62] and interruption of this association through cell cycle dependent kinase leads to proteasomal degradation of UHRF1 in M phase [16]. Zang and collaborators have recently suggested an identical role of TIP60 in regulating the stability of UHRF1 as it regulates the stability of DNMT1. They demonstrated that UHRF1 can be acetylated by TIP60 at the K659 which lies in preferential binding area of USP7 and this acetylation greatly hampered the association of USP7 with UHRF1 [63]. Our results showed that TIP60 interacts with UHRF1 through its enzymatic MYST domain and overexpression of TIP60 in HeLa cells led to downregulation of UHRF1 suggesting another mechanism for the regulation of UHRF1 in cells.

TIP60 is found downregulated in different types of cancers and is believed to have tumor suppressor properties as oncovirus like HPV induces proliferation and tumorigenesis by destabilizing TIP60 in cervical cancer cells [42–45, 64–66]. Downregulation of TIP60 is associated with increased metastasis, decreased DNA damage response to oncogenes and poor survival of patients while enhanced TIP60 levels counters DNMT1-SNAIL2 driven epithelial to mesenchymal transition and inhibits metastasis [67]. UHRF1 on the other hand, is known to play an oncogenic role in cancer as its high expression in cancer is often related to downregulation of tumor suppressor genes through promoter hypermethylation [52, 68]. We observed that overexpression of UHRF1-mCherry decreases the protein level of TIP60-eGFP (Fig. 1d) which might be attributed to promoter hypermethylation or the E3 ligase activity of UHRF1 through which it can ubiquitinate TIP60 and may possibly reduce the level of TIP60-eGFP inside the cells [18]. This is in agreement with our previous findings where knock down of UHRF1 through siRNA upregulated the TIP60 levels in Jurkat cells [21]. It is also reported that targeting UHRF1 and DNMT1 can affect the global methylation [69, 70] and re-expression of tumor suppressor genes [2]. Our results showed that TIP60 overexpression in HeLa cells induced downregulation of UHRF1 and DNMT1, resulting in global DNA hypomethylation.

## Conclusion

Epigenetic code replication machinery is a multi-protein complex which is actively involved in maintaining the epigenetic marks after the DNA replication. TIP60 and URHF1 are important members of this complex along with DNMT1. Here we conclude that TIP60 directly interacts with UHRF1 during the DNA replication phase of cell cycle and this interaction is dependent on the MYST domain of TIP60. Since UHRF1 interaction with TIP60 is known to perturb TIP60 mediated p53 activation, this study provides us with information to overcome this perturbation and counter the malicious transformations by utilizing the tumor suppressive role of TIP60. Finally, further investigations are required to fully decipher the dialogue within this three-way partnership involving UHRF1, DNMT1 and TIP60.

## Abbreviations

∆CRD: Chromodomain mutant
∆HAT: Histone acetyltransferase domain mutant
∆MYST: MYST domain mutant
∆ZnFr: Zinc Finger mutant
DNMT1: DNA methyl transferase 1
eGFP: Enhanced Green Fluorescent Protein
FLIM: Fluorescence Lifetime Imaging Microscopy
FRET: Fluorescence Resonance Energy Transfer
IP: Immunoprecipitation
TIP60: Tat interacting protein 60 kDa
UHRF1: Ubiquitin-like with PHD and RING Finger Domains 1
USP7: Ubiquitin specific protease 7
WB: Western Blotting
WT: Wild type
